# The Use of WhatsApp Smartphone Messaging Improves Communication Efficiency within an Orthopaedic Surgery Team

**DOI:** 10.7759/cureus.1040

**Published:** 2017-02-18

**Authors:** Prasad Ellanti, Andrew Moriarty, Fionn Coughlan, Thomas McCarthy

**Affiliations:** 1 Orthopaedic Department, Cappagh National Orthopaedic Hospital; 2 Department of Trauma and Orthopaedics, St James Hospital; 3 Department of Trauma and Orthoapedics, St James Hospital

**Keywords:** whatsapp, smartphone, communication

## Abstract

Introduction: Effective and timely communication is important for any surgical specialty to function. The use of smartphones is prevalent amongst doctors. Numerous smartphone applications offer the potential for fast and cost-effective communication. WhatsApp is a commonly used application that is free, easy to use, and capable of text and multimedia messaging. We report on the use of WhatsApp over a six month period in our unit.

Materials and Methods: WhatsApp communication between non-consultant members of an orthopaedic team over a six-month period was analysed. Both the phones and the WhatsApp application were password-protected, and patient details were anonymised. A series of 20 communications using the hospital pager system and the telephone system were also analysed.

Results: A total of 5,492 messages were sent during the six-month period and were part of 1,916 separate communication events. The vast majority of messages, 5,090, were related to patient care. A total of 195 multimedia messages were sent and these included images of radiographs and wounds. When using the hospital telephones, the length of time spent on a communication averaged 5.78 minutes and using the hospital pager system averaged 7.45 minutes. Using the WhatsApp messaging system has potentially saved up to 7,664 minutes over the study period. All participants found WhatsApp easy to use and found it to be more efficient than the traditional pager system

Conclusion: Compared to the traditional pager systems, the use of WhatsApp is easy, inexpensive, and reliable and can help improve the efficiency of communication within a surgical team.

## Introduction

Effective and timely communication is an absolute requirement for any surgical specialty to function. Various phone and pager systems are employed in every hospital to ensure timely communication. Smartphones are now commonplace amongst doctors and are relied upon. Numerous smartphone applications have the potential to provide fast and cost-effective communication. WhatsApp is one such application that is commonly used and, with its group communication feature, is very practical. We monitored and analysed the use of WhatsApp over a six-month period in our unit and compared it to the more traditional hospital pager and phone systems.

## Materials and methods

The WhatsApp (WhatsApp Inc., Mountain View, CA, USA) is a free cross-platform application that can be used in most smartphones and, as such, no additional equipment is required. With this application, one is able to exchange messages easily with a specific team member or the entire group and can indicate if the message has been received and read. The application is simple to use and is capable of sending multimedia messages, such as images, videos, and files as attachments, with ease. A specific “orthopaedic group” was created, which included all the non-consultant orthopaedic team. The team included five registrars, one senior house officer, and three interns. The group was used for rapid communication of work-related messages and queries amongst the team. All communication was double encrypted with a password required for access to the smartphone as well as the WhatsApp messaging application. All patient-related data was anonymised, and we used medical record numbers or bed numbers to specify patients. All WhatsApp messages used over a six-month period were included in this study for analysis. The number, types of messages, and their purpose were noted. A series of 20 communications over the hospital telephone system as well as the pager system were analysed. All the communication data was deleted from each participant’s phone at the end of the six months. At the end of the study period, we asked the team members if they found using Whatsapp to be efficient compared to traditional phone/pagers, whether it was easy to use, and if they found its use a distraction or interrupted their work.

## Results

All members of the team were familiar with and already using WhatsApp on a day-to-day basis. A total of 5,492 messages were sent during the six-month period with an average of 915 messages per month. These messages were part of the 1,916 separate communication events and involved one to four participants in any one communication. Table 1 lists the messaging activity of the team over the six-month period. The vast majority of messages (5,090 - 92.7%) were related to patient care. This included updates on new admissions, results of investigations, the progress of inpatients, and their treatment options. A further 269 (4.9%) messages were administrative, dealing with staffing and schedule changes, etc. while the remaining 133 (2.4%) messages were related to academic activities, such as teaching and research meetings. A total of 195 (3.5%) multimedia messages were sent and these included images of radiographs and wounds (Table 2). The vast majority of the communications were between the registrars and interns. Excluding messages sent outside of normal working hours (Monday to Friday, 8 am to 5 pm), most messages got a reply within seconds and most conversations were concluded within 2 minutes. Messages sent outside of working hours were largely administrative and not requiring a response.

**Table 1:** WhatsApp Messaging Activity of the Team Over the Six-Month Study Period

	Registrar n = 5	Senior House Officer n = 1	Intern n = 3	Total
Messages	3,548 (64.6%)	61 (1.1%)	1,883 (34.3%)	5,492
Average	709.6	61	627.7	
Multimedia Messages	119 (61%)	9 (4.6%)	67 (34.4%)	195
Average	23.8	9	22.3	

**Table 2:** Purpose of Messages

	Patient care (%)	Administration (%)	Academic (%)	Total
Messages	5,090 (92.7%)	269 (4.9%)	133 (2.4%)	5,492

A series of 20 random communications using the hospital telephone system and 20 communications using the pager system were monitored. When using the hospital telephones, the length of time spent on a communication averaged 5.78 minutes and using the hospital pager system averaged 7.45 minutes (Table 3). This did not include the time one had to wait for or look for an available phone. We estimate that an average of 2.5 minutes per communication would have been lost if the hospital phone system was used and an average of 4 minutes if the hospital pager system was used compared to using WhatsApp. Using the WhatsApp messaging system has potentially saved between 4,790 minutes to 7,664 minutes over the study period, depending on the modality used.

**Table 3:** Time Taken to Make Calls Using the Hospital Telephone and Pager System

	Hospital Telephone (range)	Hospital Pager (range)
Average time, mins (n = 20)	5.78 (3 - 12.5)	7.45 (3.5 - 14)

All participants felt that the use of WhatsApp was extremely useful and that it saved them a significant amount of time and effort compared to the traditional phone or pager system. All participants felt it was not a hindrance or a distraction, with the overall perception being that it freed up more time for clinical duties.

## Discussion

The relentless forward march of technology is evident in all aspects of modern life and so, too, in the hospital setting. The advent of smartphones and improved connectivity has made communication more effortless than before. Their use is prevalent amongst doctors, with studies reporting between 74% - 85% of doctors using a smartphone [1-2]. Smartphones are preferred over the traditional hospital pager systems, which are no longer considered to be efficient [3].

Johnston, et al. [4] analysed over 1,100 hours of communication amongst emergency surgical teams using WhatsApp and concluded that it was a safe and efficient form of communication. The participants of that study found WhatsApp to be an efficient tool for reducing communication barriers between senior and junior colleagues. Kelahmetoglu, et al. used WhatsApp to send images of radiographs and videos of CT scans to obtain rapid nighttime consultation on maxillofacial traumas [5]. Our study was intradepartmental, with all participants finding the use of WhatsApp made communication easier and quicker. The registrars felt that they had more regular updates on their patients than before while the interns felt that they were more supported with their decision-making as a result. With potentially more than 7,600 minutes saved on communication, all team members noted they had more time to perform clinical duties and participate in academic activities.

Wani, et al. [6] reported that the use of WhatsApp resulted in an earlier start for the management of their patients in the plastic and reconstructive surgery unit. Similarly, Astarcioglu, et al. [7] have reported earlier percutaneous coronary intervention in patients with an ST-segment elevation myocardial infarction (STEMI) in a rural setting. Their study involved 108 patients whose electrocardiograph images were sent to the interventional cardiologist using WhatsApp. This also resulted in a reduction in the false STEMI rate.

The multimedia messages sent in our study were mostly images of wounds and radiographs. The images of wounds of concern were taken at the time of the wound review by the intern and sent to the registrar for management advice. Prior to the use of WhatsApp for this purpose, the dressing would have to be taken down again for a review later during the day. This resulted in multiple exposures of the wound and potentially delayed reviews. Postoperative radiographs were sent by the interns prior to discharging patients; this had the effect of reducing delays in discharge. Preoperative radiographs were sent between registrars to inform the relevant teams of new admissions and for opinions on treatment options from senior colleagues. Giordano, et al. reported that radiographs can be sent on WhatsApp with excellent inter- and intraobserver agreement in the assessment of tibial plateau fractures and advocated expanding its use in the clinical setting [8].

Having an easily accessible form of communication can lead to increased use and to potential problems, such as frequent interruptions from the messages being sent back and forth, which could result in the likelihood of an error occurring. A review by Rivera-Rodriguez and Karsh [9] concluded that while frequent interruptions can increase the likelihood of errors, not all interruptions had a negative effect and may even be necessary for the practice of safe healthcare. None of our participants felt that WhatsApp use caused significant interruptions to performing clinical duties, and most thought the ease and speed of communication helped promote better care for their patients.

Concerns regarding the safety of transmitting confidential patient-related data over instant messaging applications have been raised [10-11]. With patient data anonymised and password access to both the smartphone and the WhatsApp application, this should minimise the risk and safeguard patient confidentiality. Furthermore, in April 2016, the WhatsApp Company introduced end-to-end encryption, which means that messages are available to the sender and receiver only, and third parties, including the WhatsApp Company itself, will not have access. Ultimately, all forms of communicating patient-related data carry the risk of breaching patient confidentiality, and it is up to the individual practitioner to sufficiently anonymise the data. While we agree that the potential for misuse of such technology exists, we feel these concerns should not limit the development and use of novel technologies within healthcare.

## Conclusions

Compared to the traditional pager systems, the use of WhatsApp is easy, inexpensive, and reliable. We feel that it can help improve the efficiency of communication within a surgical team. 
